# Supplementary material for: The Role of microRNA in the Regulation of Cortisol Metabolism in the Adipose Tissue in the Course of Obesity
Source: Int J Mol Sci. 2024 May 7;25(10):5058. doi: 10.3390/ijms25105058 (PMC11120731; doi:10.3390/ijms25105058)

Supplementary Table S1. Selected clinical parameters of study participants.

	**Obese individuals before weight loss (N = 75)**		**Obese individuals after weight loss (N = 19)**		**Normal-weight Controls (N = 25)**	
Males/Females	13/62		4/15		5/20	
	**Mean ± SD**	**Min–Max**	**Mean ± SD**	**Min–Max**	**Mean ± SD**	**Min–Max**
Age (years)	41.43 ± 10.24	20–62	41.47 ± 10.27	28-67	47.7 ± 13.53	23–62
Weight (kg)	131.15 ± 20.8	99.0-198.60	76.11 ± 7.15	68.0-90.0	67.5 ± 10.78	52.0–90.0
BMI (kg/m^2)	46.27 ± 5.51	35.43–59.52	27.2 ± 2.35	24.30-29.51	23.28 ± 1.65	20.1–24.93
Adipose tissue (% body mass)	47.09 ± 5.16	32.64-59.52	30.5± 3.35	24.8-34.05	–	–
Waist circumference (m)	1.24 ± 0.18	0.97–1.67	0.90 ± 0.12	0.78-1.05	–	–
Weight loss (kg)	–	–	47.8 ± 10.4	35.2-65.6	–	–
**Obesity-related co-morbidities**						
	N	%	N	%	N	%
Type 2 diabetes/prediabetes*	25	33.3	3	15.8	none	none
Hypertension	42	56.0	6	31.6	none	none
Hyperlipidemia	46	61.3	6	31.6	none	none
Metabolic syndrome**	40	53.3	none	none	none	none

BMI—body mass index calculated as weight (kg) divided by height squared (m^2); * impaired fasting glucose and/or impaired glucose tolerance; ** the metabolic syndrome was diagnosed based on the International Diabetes Federation criteria for Europeans: waist circumference greater than 80 cm in women and 94 cm in men, and the presence of at least 2 of 3 disorders: dyslipidemia, hypertension, carbohydrate intolerance

Supplementary Figure S1

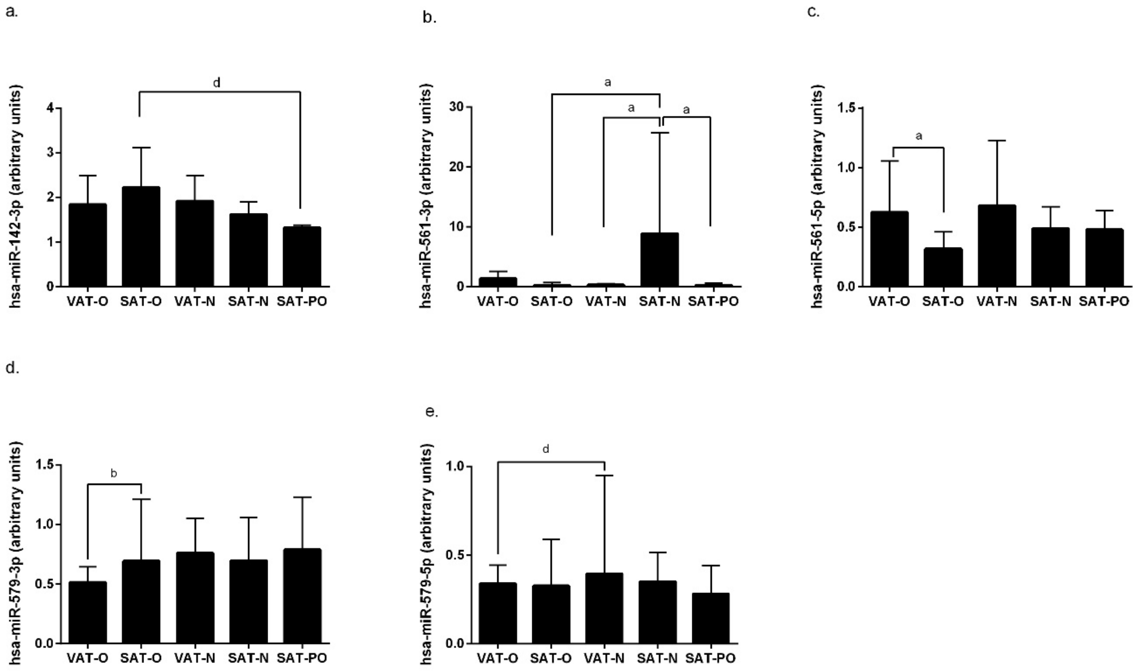

Supplementary Figure S2

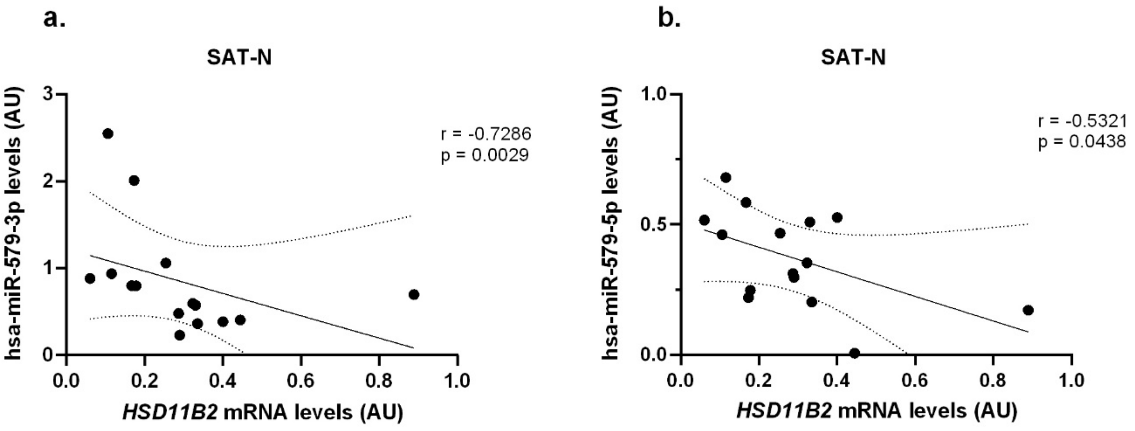

Supplementary Figure S3

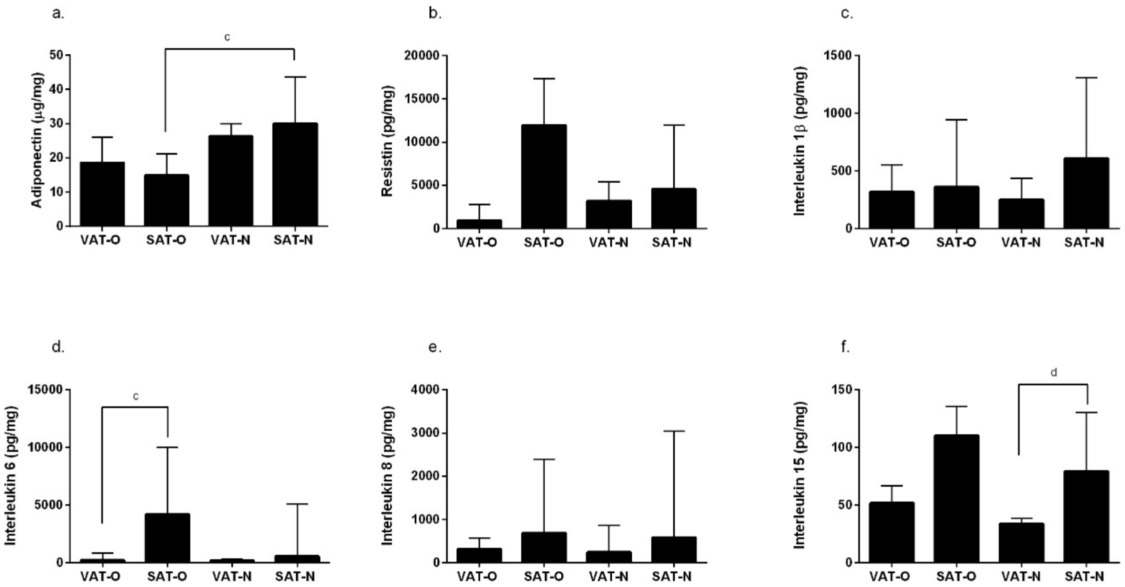

Supplementary Figure S4

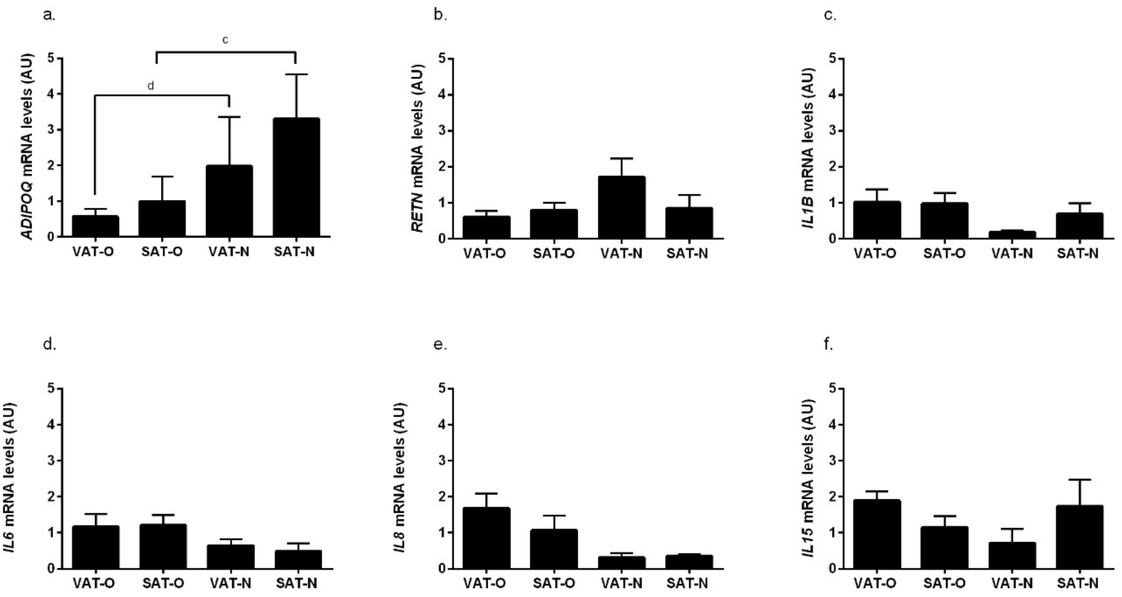

Supplement: Supplementary file 1 [file ijms-25-05058-s001.zip › ijms-2967544-supplementary.pdf]
